# Recycling industrial food wastes for lipid production by oleaginous yeasts *Rhodosporidiobolus azoricus* and *Cutaneotrichosporon oleaginosum*

**DOI:** 10.1186/s13068-022-02149-3

**Published:** 2022-05-14

**Authors:** Silvia Donzella, Immacolata Serra, Andrea Fumagalli, Luisa Pellegrino, Giacomo Mosconi, Roberto Lo Scalzo, Concetta Compagno

**Affiliations:** 1 Department of Food, Environmental and Nutritional Sciences (DeFENS), University of Milan, via L. Mangiagalli 25, 20133 Milan, Italy; 2 Present Address: Department of Biotechnology and Biosciences, University of Milan Bicocca, Piazza della Scienza 2, 20126 Milan, Italy; 3 Department of Veterinary Medicine and Animal Sciences, University of Milan, via dell’Università 6, 26900 Lodi, Italy; 4 Consiglio per la Ricerca in Agricoltura e l’Analisi dell’Economia Agraria at Centro di Ricerca Ingegneria e Trasformazioni Agroalimentari (CREA-IT), via G. Venezian 26, 20133 Milan, Italy

**Keywords:** Microbial lipids, Oleaginous yeasts, *Rhodosporidiobolus azoricus*, *Cutaneotrichosporon oleaginosum*, Food waste, Yeast biomass

## Abstract

**Background:**

Microbial lipids have been emerging as a sustainable alternative to vegetable oils and animal fat to produce biodiesel and industrial relevant chemicals. The use of wastes for microbial processes can represent a way for upgrading low value feedstock to high value products, addressing one of the main goals of circular economy, the reduction of wastes by recycling. Two oleaginous yeasts, *Rhodosporidiobolus azoricus* and *Cutaneotrichosporon oleaginosum*, were used in this study to demonstrate the feasibility of the proposed approach.

**Results:**

In this study wastes from industrial food processing, as pumpkin peels and syrup from candied fruits manufacture, were used for yeast cultivation and for lipids production. Evaluation of growth and sugar consumption revealed marked differences between the yeasts in capacity to utilize the main sugars present in the feedstock. In particular, we observed an unexpected limitation in glucose metabolism on mineral defined media by *R. azoricus*. Both species showed ability to grow and accumulate lipids on media exclusively composed by undiluted pumpkin peel hydrolysate, and *R. azoricus* was the best performing. By a two-stage process carried out in bioreactor, this species reached a biomass concentration of 45 g/L (dry weight) containing 55% of lipids, corresponding to a lipid concentration of 24 g/L, with a productivity of 0.26 g/L/h and yield of 0.24 g lipids per g of utilized sugar.

**Conclusions:**

Wastes from industrial food processing were sufficient to completely support yeast growth and to induce lipid accumulation. This study provides strong evidence that the concept of valorisation through the production of lipids from the metabolism of nutrients present in agro-industrial wastes by oleaginous yeasts is promising for implementation of biotechnological processes in a circular economy contest.

**Supplementary Information:**

The online version contains supplementary material available at 10.1186/s13068-022-02149-3.

## Background

Nowadays, the market is facing an increasing demand of triacylglycerols (TAGs) due to their application in a variety of industrial sectors. Depending on their composition, TAGs can be used not only for production of biodiesel, but also of other high value-added products, such as to cite some, biosurfactants, detergents, solvents, lubricants, adhesives, cosmetics, and degradable polymers [1–5]. In addition to chemical applications, microbial lipids can be used also as components for animal feed [6, 7].

TAGs can be obtained by vegetable oils and animal fat, but their supply cannot be sustained only by these sources, due to the large land surfaces required for cultivation and the competition to food production (the so-called fuel vs food conflict). For these reasons, more sustainable alternatives are continuously sought, and, in this context, microbial lipids (or single cell oils, SCOs) are considered as promising ones. They show a composition similar to vegetable oils and can be produced independently from geographic location, season changes, climate and by faster and controllable processes [5, 8, 9].

SCOs are produced by oleaginous microorganisms that possess the unique ability to accumulate lipids, especially TAGs, over 20% and up to 70% of the dry cell mass. Known oleaginous yeasts belong to the genera *Lipomyces*, *Yarrowia*, *Cryptococcus*, *Trichosporon* as well as *Rhodotorula* and *Rhodosporidium* [10, 11]*.* Several parameters have been reported to influence lipid production, such as pH, oxygenation, temperature, phosphate and sulfate limitation, but the most crucial one is the ratio between carbon and nitrogen (C/N ratio) in the growth medium [12, 13].Typically, when in the medium nitrogen level is low and the sugar content is still high (high C/N ratio), the carbon flux is diverted towards fatty acid biosynthesis, starting from acetyl-CoA (via citric acid) [10, 14]. Remarkably, given their increasing role played in the field of microbial oils production, some oleaginous species have been engineered to by-pass strict metabolic regulation mechanisms, to enlarge their substrate range, as well as to modify their FAs composition [11, 15–17]. Among oleaginous yeasts, some species show ability to grow using a variety of carbon sources, including those present in waste streams from the agro-food sector [18, 19]. For example, yeast-based production of oils has been reported using, among others, a wide range of lignocellulose wastes, such as dried sorghum stalks [20], cardon stalks [21], wastes generated from oil palm industries [22], mixed vegetable waste [23], but also cheese whey [24]. The generation of agro-food wastes is constantly rising due to rapid growth of world economics and population. In this regard, waste management is becoming a major concern, triggering the idea that wastes can be turned into a resource. Hence, economy has been increasingly shifted from linear to circular, with the aim to pursuit the ambitious goal of zero waste by conversion of wastes into energy and high value products. Suitable feedstock is characterized by low or null cost on the market, and this represents one of the main reasons that increase attractiveness of recycling. On the other hand, one of the principal costs that need to be addressed is related to the transport of the waste from the site, where it is generated. Another important aspect related with costs is that utilization of wastes for microbial cultivation often involves physical and/or chemical pretreatments as well as enzyme processes, that are necessary for the release of sugars available for metabolism. Toxic compounds, including organic acids, aldehydes, and phenols can be generated during harsh pretreatments and can inhibit the growth, but detoxification methods are again costly. Conversely, wastes-based media often do not require supplementary ingredients, because they result already rich in macro- and micro-nutrients necessary to support microbial growth. This can reduce the costs of the process.

In this paper we explored the valorization of wastes derived from the food industry, in particular pumpkin peels. Pumpkins (family *Cucurbitaceae*) are consumed in many ways, in soups, juices, or incorporated into various foods, such as cakes, candies and bread. They are rich of proteins, antioxidant vitamins, such as carotenoids and tocopherols, and minerals, but low in fat and calories. Species such as *Cucurbita maxima*, *Cucurbita moschata*, and *Cucurbita pepo* are planted in temperate and subtropical regions over the world, and Italy is among the main world producers, being placed ninth according to the FAO (Food and Agriculture Organization of the United Nations) statistics [25]. By setting up a protocol of enzymatic pretreatments, we showed the feasibility of producing yeast biomass rich of lipids from the metabolism of compounds present in pumpkin peel hydrolysate, using the oleaginous yeasts *Rhodosporidiobolus azoricus* (previously known as *Rhodosporidium azoricum*) and *Cutaneotrichosporon oleaginosum* (previously known as *Trichosporon oleaginosus*). Yeasts were cultivated on media entirely derived from pumpkin peel wastes without any addition of nutrients. To increase lipid accumulation, a two-stage process was carried out in bioreactor, using another food waste, a syrup derived from candied fruits manufacture, rich of available sugars that don’t need any pretreatment. In conclusion, this study provides strong evidence that the concept of valorization of agro-industrial wastes by oleaginous yeasts is promising, for implementation of biotechnological processes in a circular economy contest.

## Results and discussion

### Optimization of pumpkin peel waste pretreatments and quantification of components

Pumpkin cultivar can influence physical properties of the waste such as density, rheological aspects and can also greatly affect chemical composition [26]. Components of different pumpkin cultivar include organic acids and soluble sugars, mainly fructose, glucose, and sucrose, giving typical tasty traits [27], but presence of starch is also reported [28, 29]. Pumpkin peel waste utilized in our study was composed by a tiny peel, probably containing cellulose/hemicellulose, but some residual pulp was present. For these reasons, enzymatic hydrolysis was considered as pretreatment to increase the amount of sugars available for fermentation. Commercial preparation containing a blend of cellulases, ß-glucosidases, and hemicellulases (Cellic CTec2) was tested to optimize the amount of enzyme and time of hydrolysis suited for pumpkin peels treatment. Different amounts of enzyme blend were added to the feedstock (details reported in “Methods” section). We observed that during the enzymatic treatment, the biomass became more broken up, and a minimum of 24 h was required to obtain an almost liquefied mixture. The reactions of hydrolysis were monitored by quantifying the released glucose (Table 1). The addition of 2.25 μL/mL of enzyme blend allowed the release of 23.6 g/L of glucose in 24 h (Table 1), and higher concentration of enzyme did not release more. Since glucose concentration did not increase after further 24 h (48 h from the start), this time was considered optimal for hydrolysis. On the other hand, sucrose and fructose concentrations remained constant over the time at all the tested enzyme concentrations (sucrose 29 g/L, fructose 5 g/L,). Therefore, these conditions (enzyme blend 2.25 μL/mL, 24 h) were selected to obtain pumpkin peel waste hydrolysate suitable for fermentative processes. Due to the known presence of starch among the pumpkin components, we tested also if addition of commercial amylase (A8220 from *Aspergillus oryzae*), together with the cellulose blend, could increase the amount of glucose in the hydrolysate, but we did not obtain this result. This was due to the presence of amylase activity in the commercial cellulolytic blend (Cellic CTec2), as we tested using starch as substrate (data not shown). The enzymatic hydrolysate obtained after 24 h was characterized for its composition (Table 2). Analysis by HPLC showed that sucrose was the principal sugar found in the soluble fraction (fraction 1), (Table 2, Additional file 1: Table S1), together with glucose and fructose. Sucrose is mostly present in the pulp, thus it can be found at different levels in the wastes, depending on the type of peeling operation adopted in industrial processing. Oligosaccharides at different molecular weights (DP4 and DP3) were also found in fraction 1 (Additional file 1: Table S1). The presence of unbound galacturonic (Table 2, Additional file 1: Table S1), suggested that pectines are also among the components of pumpkin peel waste. Glucuronic acid was also found. The presence of only 25 mg/g of glucose in fraction 2 (Additional file 1: Table S1), indicated that most of the starch had been hydrolyzed by Cellic CTec2 treatment. The absence of sugars in fraction 4 (Additional file 1: Table S1) confirmed that cellulose/hemicellulose in the pumpkin peel waste had been completely hydrolyzed by Cellic CTec2 treatment. Quantification of acetic acid (Table 2) was also done, because it is known to be an inhibitor of cell growth.

**Table 1:** Enzymatic pre-treatments performed on pumpkin peel waste (small scale)

Cellic CTec2 (µL/mL)	24 h-Glucose (g/L)	48 h-Glucose (g/L)
–	3.9 ± 0.3	4.1 ± 0.5
1.12	17.2 ± 0.4	19.2 ± 0.3
2.25	23.6 ± 0.5	23.8 ± 0.6
5.5	22.3 ± 0.5	22.7 ± 0.8
11.25	21.8 ± 0.7	20.8 ± 0.6
22.5	21.1 ± 0.6	19.3 ± 0.7

Different amounts of enzyme were tested. Glucose concentrations obtained after 24 h and 48 h of hydrolysis are reported

**Table 2:** Concentration of the main components of pumpkin peel hydrolysate (after 24 h of enzymatic digestion, large scale)

Medium component	Concentration (g/L)
Glucose	18 ± 1.52
Sucrose	29 ± 1.80
Fructose	5.0 ± 0.60
Xylose	0.4 ± 0.08
DP6–DP3	9.0 ± 0.93
Acetic acid	0.9 ± 0.20
Galacturonic acid	8.0 ± 0.09
Glucuronic acid	6.5 ± 0.12
Nitrogen	2.4 ± 0.31

Nitrogen (N) is an essential element for biomass production; nevertheless, lipid production is triggered by high C/N ratio [10]. When the C/N ratio is low the cells invest nitrogen and carbon to produce biomass, whereas when the ratio is high the carbon is mainly directed toward lipid production. In the pumpkin peel hydrolysate inorganic nitrogen (NH_4_^+^) was found only in traces, but quantification of total nitrogen accounted for 2.4 g/L, then revealing that it contains mainly organic N (Table 2).

### Analysis of growth and sugar utilization on mineral defined media

The availability of a pumpkin peel hydrolysate containing sucrose, glucose and fructose as principal sugars lead us to evaluate first the capacity of *R. azoricus* and *C. oleaginosum* to grow in their presence. Cultures were performed on mineral defined medium (YNB), to avoid the utilization of other carbon sources that could mask the ability to consume provided sugars. In this way, we could also obtain quantitative data, in terms of sugar consumption rate and biomass yield. These parameters are known as key factors for selecting suitable species and for developing efficient fermentation processes [30].

**Fig. 1:**
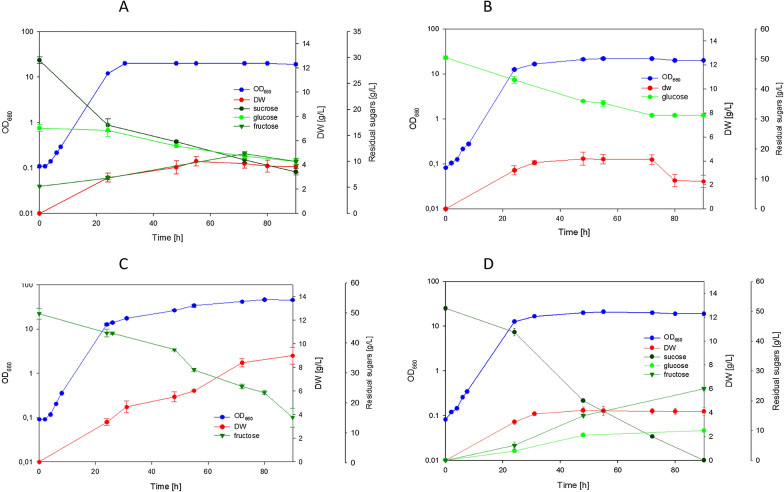
Cultivation of *R. azoricus* on YNB medium containing: **A** mixture of 30 g/L sucrose, 15 g/L glucose and 5 g/L fructose; **B** 50 g/L of glucose; **C** 50 g/L of fructose; **D** 50 g/L of sucrose

**Fig. 2:**
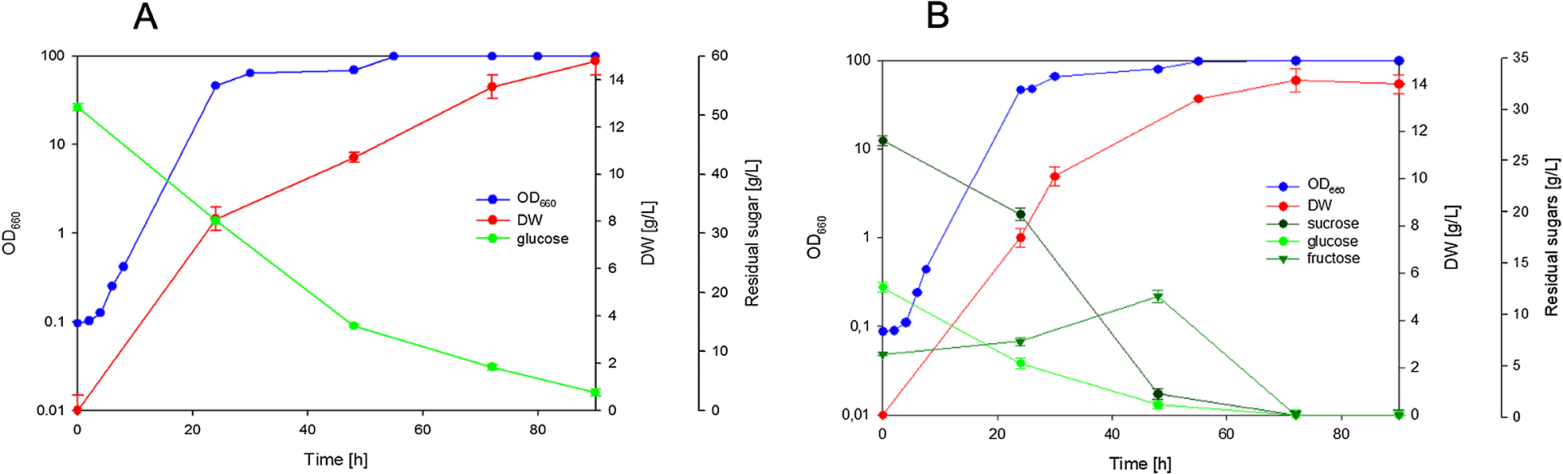
Cultivation of *R. azoricus* on YNB medium supplemented with 1 g/L of yeast extract: **A** YNB-glucose medium; **B** YNB-mixture medium, 30 g/L of sucrose, 15 g/L of glucose, 5 g/L of fructose

The cultivation of *R. azoricus* on YNB containing a mixture of sucrose, glucose and fructose, at concentration similar to pumpkin hydrolysate, showed that this yeast starts to hydrolyze sucrose even in presence of other available sugars (Fig. 1A). The increase of fructose concentration after 24 h suggested extracellular localization of invertase activity necessary for sucrose hydrolysis, similarly to what reported in *C. curvatus* [31]. However, a limited capacity to utilize the derived glucose and fructose was observed. After 72 h both sugars were partially assimilated (Fig. 1A), and yeast biomass reached a concentration of 4 g/L of dry weight, with a low yield (Table 3). We did not found production of other compounds coming from sugar metabolism, such as ethanol, lactic acid or acetic acid. Cellular viability was then tested, and we found that 30% of cells were not viable after 72 h. In the light of these results, we analyzed the growth in presence of single sugar as sole carbon source. When the cells were cultivated on glucose (Fig. 1B), *R. azoricus* started to grow exponentially, but we observed an early arrest in consumption of this sugar; after 72 h only 20 g/L were consumed, producing again a low concentration of yeast biomass (4 g/L) with a low yield (Table 3). Cellular viability was tested and, surprisingly, also in this case we found that 40% of cells were not viable after 72 h. A partial utilization of glucose was observed also by decreasing the initial glucose concentration from 50 to 25 g/L (data not shown). By contrast, the cultivation on sole fructose revealed that this sugar did not cause any early arrest of growth (Fig. 1C). By comparing growth parameters on glucose and on fructose containing media (Table 3), it was evident that, despite glucose specific consumption rate was higher than fructose, the growth rates were rather similar (Table 3). The main differences were found in terms of final amount of produced biomass (4 g/L on glucose and 9 g/L on fructose) and on biomass yield (0.2 and 0.3, respectively), indicating that fructose was metabolized in a more efficient way than glucose. However, cultivation on YNB-sucrose (Fig. 1D) showed that the cells were able to hydrolyze sucrose, but the released monomers glucose and fructose were partially metabolized, leading to production of low concentration of biomass (Table 3). These results suggest that the presence of glucose in the medium, even when released by sucrose hydrolysis, can cause a negative effect on sugar utilization and early arrest of growth with low production of biomass. This limited ability by *R. azoricus* to metabolize glucose observed on mineral defined media (YNB) was unexpected, because it had been previously documented that *R. azoricus* grows efficiently on glucose-based media used for lipid production [17, 32, 33]. However, those media contained yeast extract or corn steep, and were not exclusively mineral like the one used in the present study. On the other hand, the commercial YNB contains all the compounds necessary for the growth, in the form of salts, vitamins and trace elements. This prompted us to investigate if addition of yeast extract can improve sugar metabolism. By supplementing yeast extract to YNB-glucose cultures (Fig. 2A) as well as to YNB-sucrose cultures (not shown), a positive effect on growth and on sugar utilization was observed. The same positive effect was exerted also in cultures with sugar mixture (Fig. 2B): after 72 h, sucrose was completely hydrolyzed, and glucose and fructose depleted, leading to production of 14 g/L of biomass. These results demonstrate that enrichment of the mineral defined medium by other nutrients, contained in yeast extract but not in YNB, is very important for sugar metabolism in this yeast species. This beneficial effect was, therefore, at the basis of the efficient growth on glucose containing media previously utilized for lipid production processes [17, 32, 33]. Recently it has been reported such a positive role of supplementing amino acids on metabolism of all the carbon sources present in sugar beet pulp hydrolysates by *Rhodotorula* strains [34].

**Table 3:** Growth parameters of *R. azoricus* and *C. oleaginosum* cultivated in shaken flasks on YNB media containing different carbon sources

	*R. azoricus*					*C. oleaginosum*				
	µ_max_ (h-1)	q (mmol_sug_/g_dw_/h)	Biomass yield (g_dw_/g_sug_)	Final biomass (g/L)	Consumed sugars (g/L)	µ_max_ (h-1)	q (mmol_sug_/g_dw_/h)	Biomass yield (g_dw_/g_sug_)	Final biomass (g/L)	Consumed sugars (g/L)
Glu + Fru + Suc	0.22 ± 0.02	ND	0.22 ± 0.07	4.10 ± 0.42	18 ± 1.32	0.26 ± 0.08	ND	0.32 ± 0.08	12.06 ± 0.46	37 ± 1.76
Glucose	0.21 ± 0.02	3.40 ± 0.09	0.20 ± 0.04	4.00 ± 0.39	20 ± 1.00	0.22 ± 0.09	3.21 ± 0.21	0.33 ± 0.09	12.00 ± 0.51	36 ± 1.30
Fructose	0.23 ± 0.03	2.51 ± 0.17	0.32 ± 0.07	9.01 ± 0.21	28 ± 1.50	ND	ND	ND	ND	ND
Sucrose	0.22 ± 0.05	ND	0.25 ± 0.06	3.90 ± 0.22	15 ± 2.23	ND	ND	ND	ND	ND

*ND* not determined

**Fig. 3:**
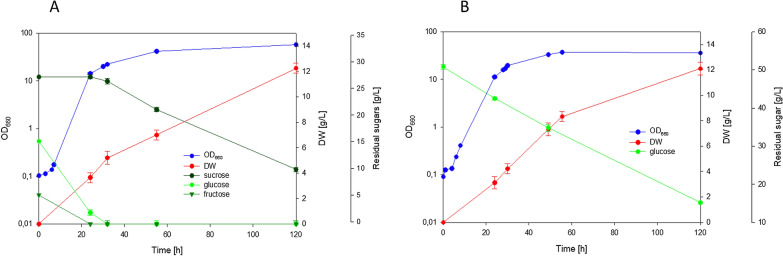
Cultivation of *C. oleaginosum* on YNB medium containing: **A** mixture of 30 g/L sucrose, 15 g/L glucose and 5 g/L fructose; **B** 50 g/L of glucose

When *C. oleaginosum* was cultivated on YNB containing mixture of sugars (glucose, fructose and sucrose) we observed a different behavior. The cells initially grew using already available monosaccharides, glucose and fructose (Fig. 3A). After their depletion, sucrose was hydrolyzed, indicating that the presence of free sugar monomers delayed sucrose hydrolysis. After 120 h, sucrose (10 g/L) was still present in the medium. However, in this culture a higher amount of biomass was obtained (12 g/L) in comparison to *R. azoricus* culture, and with a higher yield (Table 3), due to the complete utilization of monosaccharides. On the other hand, cultivation on YNB containing glucose as sole carbon source (Fig. 3B) showed an increase of biomass production consequent to glucose utilization, indicating that in this yeast any early arrest of growth caused by this sugar occurs, in contrast to what observed in *R. azoricus*.

By comparing the results obtained in terms of final biomass concentration and yield (Table 3), we can conclude that *C. oleaginosum* is able to use glucose on mineral defined media in a more efficient way than *R. azoricus*. However, it is noteworthy that the metabolic problem relative to glucose utilization by *R. azoricus* can be eliminated by the addition of yeast extract or peptone. These results reinforce the necessity of studies that carefully analyze growth parameters to identify the basis of metabolic diversity among oleaginous yeasts, as recently reported [35, 36]. This kind of information is often lacking, because mineral defined media are not suitable for industrial applications. The limited ability to metabolize glucose by *R. azoricus* and its early cellular death could lead to think to a phenomenon of glucose toxicity. In mammalian cells under hyperglycemic conditions, activation of the aldose reductase pathway causes redox unbalance and induction of oxidative stress, upregulating glucose toxicity pathways, as non-enzymatic glycation and disruption of mitochondrial respiratory chain [37]. Aldose reductases are present in yeasts and have been studied mainly for the production of sugar alcohols [38, 39], but their role in regulation of sugar metabolism has been scarcely investigated.

### Biomass and lipids production from pumpkin peel waste utilization

With the aim of developing fermentative processes for the production of biomass and lipids from pumpkin peel wastes, shaken flask cultures of *R. azoricus* and *C. oleaginosum* were set up. For this purpose, we tested the possibility to utilize for yeast cultivation a medium exclusively derived from pumpkin peel hydrolysate, without any additional nutrient. This medium contains sugars and nitrogen source, and, in addition, also acetic acid (Table 2, Additional file 1: Table S1). *R. azoricus* and *C. oleaginosus* are reported not able to grow on glucuronic acid [40, 41], and *R. azoricus* can metabolize acetic acid [32].

**Fig. 4:**
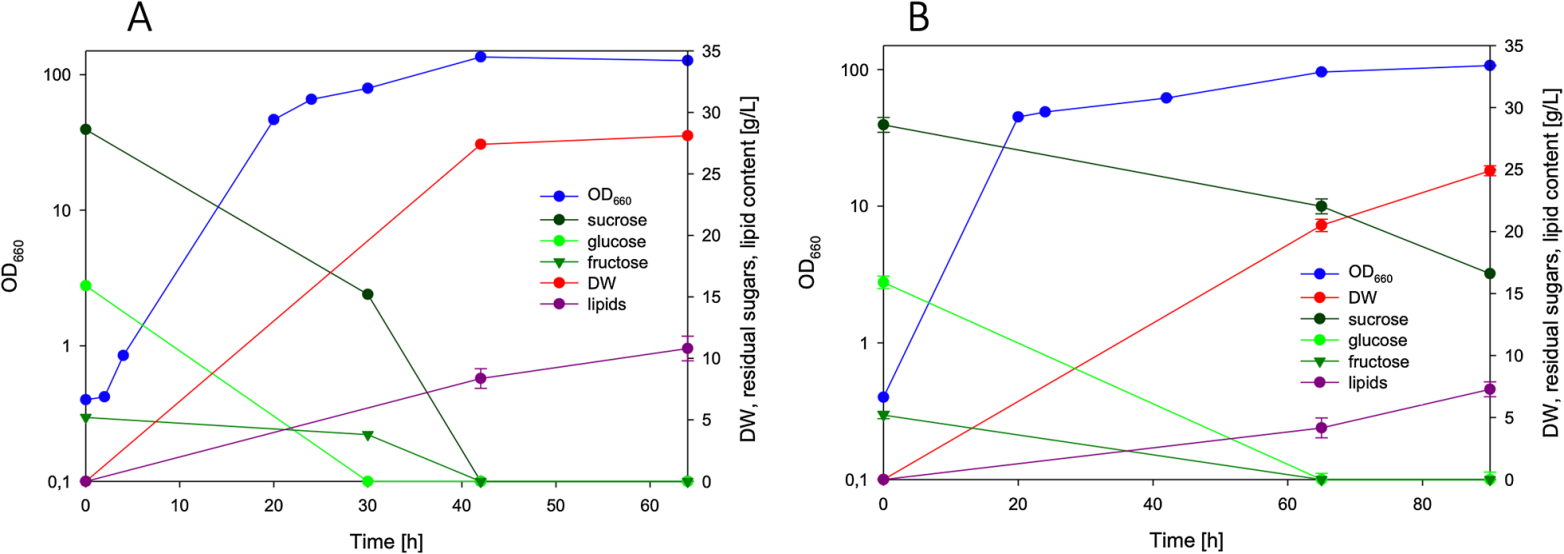
Cultivation of *R. azoricus* and *C. oleaginosum* in shaken flasks on pumpkin peel hydrolysate-based medium

After 42 h of cultivation, we observed that *R. azoricus* consumed all the main sugars (sucrose, glucose and fructose, Fig. 4A) as well as acetic acid, as expected. However, we observed that also galacturonic acid was used, analogously to what reported by *Rhodotorula toruloides* [34]. On the other hand, oligosaccharides (DP6–DP3) were partially consumed (5 g/L consumed). We can conclude that the presence of amino acids in pumpkin peel hydrolysate played a positive role for sugar metabolism, analogously to what observed by addition of yeast extract (YE) to YNB media (see paragraph above). As a consequence, and due to the higher carbon and nitrogen content of this medium in comparison to the YNB-based one, yeast biomass reached a dry weight of 28.1 g/L after 64 h. This final biomass contained 39% of lipids, corresponding to a concentration of 11 g/L.

When *C. oleaginosum* was cultivated on the same medium, sucrose was only partially hydrolyzed after 65 h (Fig. 4B), but glucose and fructose were exhausted. Acetic acid was metabolized also. These results confirmed that in *C. oleaginosum*, as observed in mineral defined medium (see paragraph above), sucrose utilization is delayed by the presence of monosaccharides, and this negative effect is not relieved by the presence of amino acids, in contrast to what observed in *R. azoricus*. As a consequence, lower levels of biomass (25 g/L) and lipids were obtained; lipids represented 29% of yeast dry weight, that corresponded to a concentration of 7 g/L.

In conclusion, these results indicated that pumpkin peel hydrolysate represents a complete source of nutrients for cultivation and lipid production by *R. azoricus* and *C. oleaginosum*. In particular, *R. azoricus* shows a natural ability to co-metabolize the contained carbon sources, that is an important trait for complex wastes utilization.

### Two-stage process in bioreactor for increased lipid production

Based on the observation that *R. azoricus* exhibited the best performance on pumpkin peel hydrolysate-based medium, we developed a fermentative process to increase lipid production. Usually, the process for lipid production is performed in two stages, the first carried out on media at low C/N ratio to produce high amount of cell biomass, and the second at high C/N ratio to trigger lipid accumulation [10].

**Fig. 5:**
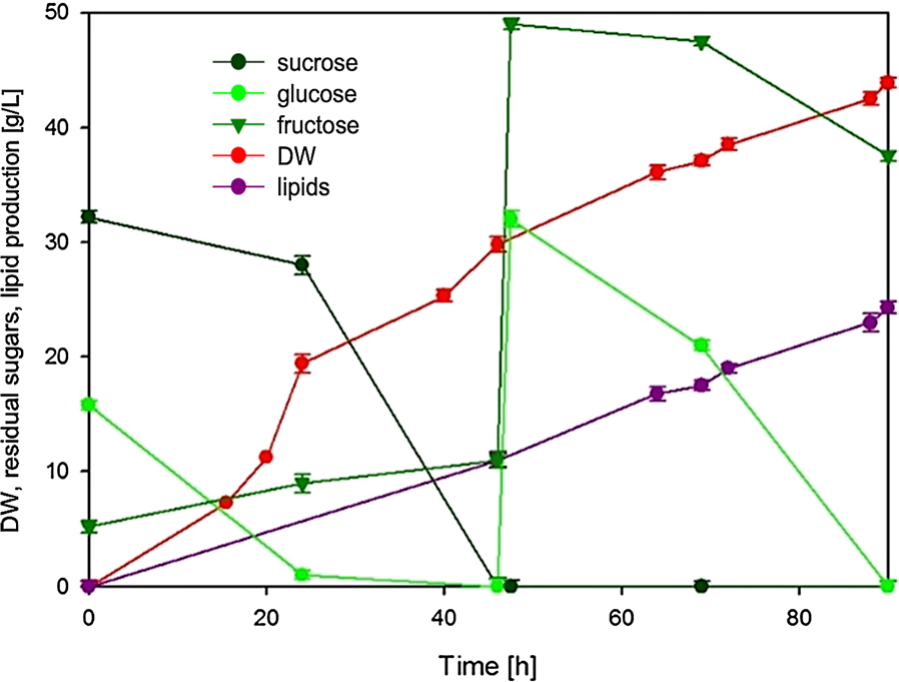
Two-stage process of *R. azoricus* on pumpkin peel hydrolysate-based medium (first stage) and by feeding with candied fruits syrup (second stage)

The cultivation in bioreactor using pumpkin peel waste hydrolysate as medium (Table 2), under controlled conditions of oxygen and pH, resulted in the production of 30 g/L of biomass, that contained 37% of lipids, after 46 h of cultivation (Fig. 5). At this point, sucrose and glucose were exhausted, whereas some fructose was still present. As observed in flask cultures, acetic acid as well as galacturonic acid were also consumed (data not shown).

With the aim to increase lipid content of the biomass, another industrial food waste coming from the manufacture of candied fruits (a syrup from mango processing) was used to feed the culture. This waste contains high concentration of available glucose and fructose (199 and 296 g/L, respectively), without the need of any pretreatment. In addition, it does not contain nitrogen, making it a perfect source of sugars to increase C/N ratio of the medium and trigger lipid accumulation. In the following 44 h of process, all glucose and part of fructose were metabolized (Fig. 5). Sugars were converted into lipids, allowing the yeast biomass to reach a dry weight of 45 g/L with a lipid content of 55%. A lipid concentration of 24 g/L was obtained after 90 h of process (Fig. 5), that corresponded to a lipid productivity of 0.26 g/L/h. Based on the consumed carbon sources the lipid yield was 0.24.

In conclusion, these results represent a very promising starting point for developing a lipid production process. By the exclusive use of food wastes (pumpkin peels and candied fruits syrup), *R. azoricus* was able to efficiently grow and produce lipids with high productivity and yield. Similar results have been reported using other oleaginous yeast cultivated on agro-food wastes. Orange peel extract supplemented with ammonium sulphate has been shown to allow production of 7.6 g/L of *Cryptococcus laurentii* biomass containing 59% of lipids and 6.9 g/L of *Rhodosporidium toruloides* containing 79% of lipids in batch cultures [24]. *R. toruloides* cultivated on Jerusalem artichoke extract has been reported to produce 40 g/L of biomass with 43% of lipids [42]. In [43] a process under pulsed-feeding cultivation on sugar beet pulp (SBP) and molasses by the oleaginous yeast *Lipomyces starkeyi* was reported. They obtained 20.5 g/L of final biomass with a lipid content of 49.2%. Other examples of lipid production from organic wastes are reported in the recent review by [38, 44].

### Lipid analysis

**Fig. 6:**
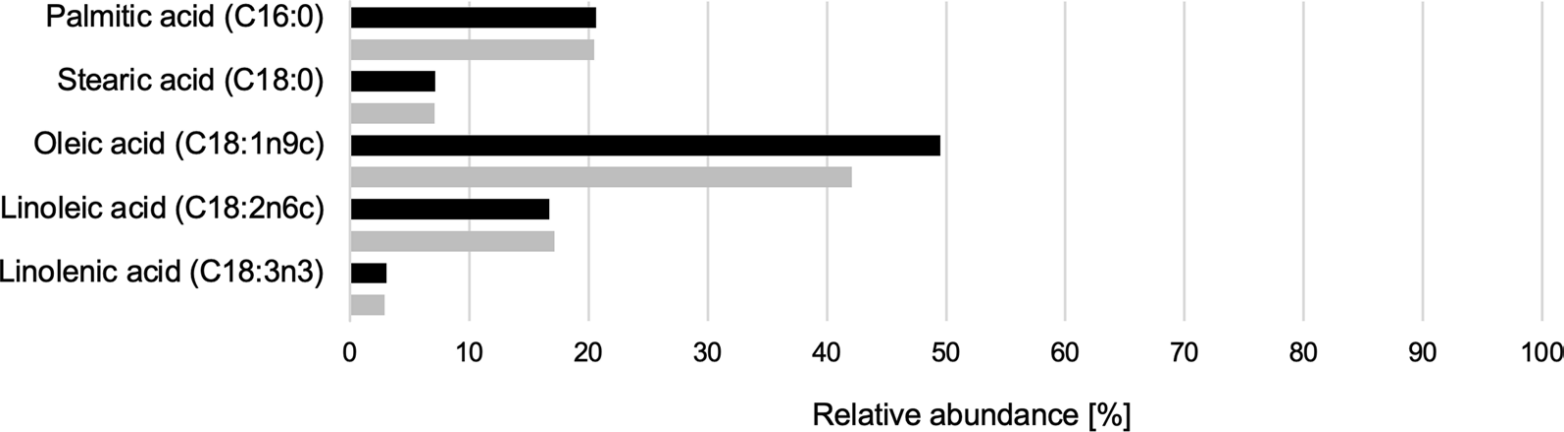
Comparision between major fatty acids produced by *R. azoricus* on pumpkin peel hydrolysate-based medium (black) and on corn stover hydrolysate [32] (grey)

Lipids synthesized by *R. azoricus* are known to be mainly composed of long-chain fatty acids with 16 and 18 carbon atoms, with a profile similar to vegetable fats [45]. At the end of the process on pumpkin peel hydrolysate-based medium, the percentages of saturated, monounsaturated and polyunsaturated fatty acids resulted 30.16%, 50.12% and 19.73%, respectively. The main fatty acids produced under this condition were: 49.53% oleic acid (C18:1), 20.65% palmitic acid (C16:0), 16.69% linoleic acid (C18:2), and a small percentage of stearic acid (C18:0, 7.15%), and linolenic (C18:3, 3.03%). In addition, we found a significant amount of omega-3 (3.03%) and omega-6 (16.69%). The complete analysis is reported in Additional file 1: Table S2. On the whole, lipid profile obtained by cultivation on pumpkin peel hydrolysate-based medium appears quite similar to those obtained on a lignocellulosic hydrolysate [32], except for a small increase in oleic acid content (Fig. 6). These results indicate that the composition of pumpkin peel hydrolysate did not significantly affect the fatty acid profile of *R. azoricus*.

### Conclusions

This proof-of-concept study has shown the feasibility to produce yeast biomass and lipids in an economical way, demonstrating a possible process of upgrading low value industrial food wastes to high value products. The use of two inexpensive residues, i.e. pumpkin peels and syrup from candied fruits processing, for sustaining microbial growth and oil production has been never reported before. These wastes represent a renewable and low-cost feedstock to fulfil nutrient demand by yeasts. In particular, *R. azoricus* valorized the pumpkin peel residues by reaching higher production of biomass and lipids, due to its better ability to hydrolyze sucrose and metabolize derived monomers contained in this waste.

Lipids can be extracted and used to produce biofuels and chemicals. Yeast biomass can in turn be used as a high-quality animal feed ingredient, because it is considered a well-balanced source of protein, and can provide also vitamins (mainly the B group) [46]. In conclusion, one of the main goals of circular economy, which is the reduction of wastes by recycle, could be addressed.

## Methods

### Strains, media and growth conditions

The yeast strains used in this work are: wild-type strain DBVPG 4620 *Rhodosporidiobolus azoricus*, also known as *Rhodosporidium azoricum* [33, 47] and wild-type strain ATCC 20509 *Cutaneotrichosporon oleaginosum* [48]. For long-term storage, yeast strains were maintained at − 80 °C on 15% (v/v) glycerol.

YPD medium contained 10 g/L yeast extract (Biolife, Italy) 20 g/L peptone (Biolife, Italy) and 20 g/L glucose (Sigma Aldrich, Italy).

YNB medium contained Yeast Nitrogen Base without amino acids and ammonium sulfate 0.17 g/L, (Difco BD, Italy), ammonium-sulfate 5 g/L and 0.1 M MES hydrate (Sigma Aldrich, Italy) to maintain pH 6. Sugars (sucrose, glucose, fructose from Sigma Aldrich, Italy) were added individually at 50 g/L, or in mixture to simulate the pumpkin peel hydrolysate composition (sucrose 30 g/L, glucose 15 g/L, fructose 5 g/L). In some experiments 1 g/L of yeast extract was added.

Yeast cells were cultivated at 28 °C in a rotary shaker at 150 rpm in bluffed flasks with an air–liquid ratio of 5:1. Cells from pre-cultures (inoculated from glycerol stocks) grown overnight on YNB-glucose were harvested by centrifugation (5000 rpm/2300 rcf) 10 min in Eppendorf 5415D centrifuge) and inoculated at OD_660_ 0.1 in YNB medium with different carbon sources or at OD_660_ 0.4 in pumpkin peel hydrolysate-based medium. Cell growth was monitored by measuring the increase of optical density at 660 nm (OD_660_) using a spectrophotometer (Eppendorf, Italy).

To determine cell viability, the cell pellet was suspended in PBS, and a sample (100 μL) of the cell suspension was mixed with 100 μL methylene blue (0.1 g/L stock solution, dissolved in a 2% dihydrate sodium citrate solution) and incubated for 5 min at room temperature. Viability was examined under microscope, since viable cells were colorless, and dead cells were blue.

### Preparation of pumpkin peel hydrolysate

Fresh pumpkin peels (from Azienda Agricola Paratorosso, Settala, Milan, Italy) were oven-dried over-night at 70 °C and milled with a laboratory grinder Bimby TM 3300 to obtain a fine powder. The powder was stored at 4 °C in a tightly closed jar.

Small scale enzymatic hydrolysis was carried by suspending the peel powder solid loading of 12% (w/v), in distilled water. The mixture was then sterilized in autoclave (Cavallo S.r.l., Milan, Italy) at 0.5 atm, 112 °C for 30 min. After cooling to 50 °C, the mixture was adjusted at pH 5.5 added with different amounts (1.12, 2.25, 5.5, 11.25, 22.5 µL/mL) of Cellic CTec2 enzyme cocktail (hydrolytic activity > 1150 U/mL) (SAE0020, Sigma Aldrich, Italy) and incubated at 50 °C in an oil bath under magnetic stirring. At different times, 0.5 mL aliquots were collected, and sugar content was analyzed by enzymatic assay.

The same procedure was applied to test α-amylase from *Aspergillus oryzae* (A8220, specific activity ≥ 800 FAU/g, Sigma Aldrich, Italy) on pumpkin peel waste. Different amounts of α-amylase (5, 10, 20, 50 µL/mL) were added and incubated at 50 °C in an oil bath under magnetic stirring. Glucose content was analyzed by enzymatic assay after 24 h of digestion.

The higher scale hydrolysis was carried out in a 2-L bioreactor (Applikon Biotechnology, The Netherlands). The bioreactor filled with the reaction mixture (650 mL) was autoclaved for 1 h at 1 atm, 121 °C. Temperature was set at 48 °C, agitation at 650 rpm and pH at 5.5. To avoid risk of bacterial contamination, antibiotics were added (Ampicillin 100 µg/mL, Kanamycin 10 µg/mL and Chloramphenicol 5 µg/mL, from Sigma Aldrich, Italy). The hydrolysis was started by the addition of enzymes (1.46 mL of Cellic CTec2). After 24 h, the hydrolysate was centrifuged in sterile tubes at 5000 rpm/3214 rcf in Eppendrof 5804R centrifuge for 1 h, and the liquid phase stored at − 20 °C.

### Fed batch cultivation

Fed-batch cultures were performed in a 2-L bioreactor (Applikon Biotechnology, The Netherlands), with a starting volume of 600 mL. Pumpkin peel hydrolysate was sterilized and used as medium for yeast cultivation after sterilization by autoclaving at 112 °C (0.5 atm) for 30 min. Temperature was set at 30 °C, the air inlet at 1 vvm and the agitation at 500 rpm. Dissolved oxygen concentration was measured by AppliSens oxygen probe (Applikon Biotechnology, The Netherlands), starting from 100% of saturation. Foam formation was controlled by the addition of a silicon antifoaming agent (Sigma 204 from Sigma Aldrich, Italy). The pH, measured by AppliSens pH electrode (Applikon Biotechnology, The Netherlands), was automatically adjusted and maintained at 5.5 by adding 5 M KOH or 10% (v/v) solution of H_2_SO_4_. Sterilized and diluted syrup from candied fruits (mango) manufacture (SVZ, Industrial Fruit & Vegetable Ingredients, Breda, the Netherlands), containing glucose 199 g/L and fructose 296 g/L, was supplied after 46 h to increase the C/N ratio of the medium.

### Pumpkin peel waste sugar analysis

Pumpkin peel waste composition analyses were performed following methods from [49] and [50]. Bravely, 500 mg of lyophilized sample obtained after pumpkin peel waste hydrolysis was dissolved in 10 mL of distilled water, treated with ultrasound for 10', centrifuged at 4950 rpm for 20′. Supernatant, cleaned by filtration on 0.45 µm nylon filters, was analyzed by HPLC (fraction 1). The pellet, suspended in 10 mL acetate buffer 0.1 M pH 4.5, was hydrolyzed by 2000 U of fungal diastase (09962, Sigma Aldrich, Italy), 100 U of alpha-amylase heat stable (A3306, Sigma Aldrich, Italy), 200 U of fructanase mixture exo and endo-inulinase (E-FRMXPD, Megazyme, Ireland), incubating at 42 °C for 2 h. After centrifugation, supernatant was cleaned and analyzed as before (fraction 2). The pellet suspended in 10 mL of acetate buffer 0.1 M pH 4.5, was added by 100 U of pectinase from *Aspergillus niger* (17389, Sigma Aldrich, Italy), 10 U of pectin-esterase from tomato (P6763, Sigma Aldrich, Italy) and incubated at 42 °C for 2 h. After centrifugation, the supernatant was cleaned and analyzed as for fraction 1 (fraction 3). The pellet suspended in 10 mL acetate buffer 0.1 M pH 4.5, added by 100 U of cellulase from *Trichoderma reesei* (C2730, Sigma Aldrich, Italy), and incubated a 42 °C for 4 h. After centrifugation, the supernatant was cleaned and analyzed as before by HPLC (fraction 4). The sugars fraction extracts were subjected to two different HPLC analyses, one to detect neutral sugars, another to detect acidic sugars.

HPLC conditions for neutral sugars: Jasco system, data acquiring system Apex Clarity version 2.6.5.517, column Benson carbohydrate, Ca^++^, 300 × 7.8 mm, 0,65 mL/min, 80 °C, mobile phase bi-distilled water, detector RI, injection volume 30 µL. For the detection, DP6-DP3 oligosaccharides (5.4–6.4 min), cellobiose (8.0), sucrose (8.5), glucose (10.3), fructose (12.5), galactose-xylose (11.3), rhamnose (12.9) and arabinose (13.5) were tentatively identified and quantified by comparison of their retention times.

HPLC conditions for acidic sugars: Agilent system, series 1200, column Repromer H^+^, Dr. Maisch, GmbH, 300 × 8 mm, 0.5 mL/min, 65 °C, mobile phase H_2_SO_4_ 25 mM, detector UV 205 nm, injection volume 30 µL. For the sugars detection, galacturonic acid (10.6 min) and glucuronic acid (13.8), were tentatively identified and quantified.

The calibration was made by external standards, with calibration curves of minimum 3 points (0.2–1.2 mg/mL). Data have been obtained in triplicate and are given as mg/g of starting substrate (Additional file 1: Table S1). The same method was used to analyze the medium at the end of fermentation processes.

### Sugars and acetic acid determination

The concentrations of sugars and acetic acid during fermentation processes were determined by employing commercial enzymatic kits (K-GLUHK and K-SUFRG, Megazyme, Ireland and 10148261035, R-Biopharm AG, Germany, respectively). All the assays were performed in triplicate and standard deviations varied between 1 and 5%.

### Nitrogen determination

Inorganic nitrogen was determined by employing a commercial enzymatic kit (10542946035, R-Biopharm AG, Germany).

Total nitrogen concentration in culture supernatants was determined by Kjeldahl method using a SpeedDigester K-376 and a KjelMaster K-375 (Buchi, Italy).

### Dry weight determination

Cells were collected from the medium (1 or 2 mL of cell culture) by centrifugation (5 min at 13,200 rpm/16100 rcf in Eppendrof 5415D centrifuge). The pellets were dried overnight at 105 °C. In the case of pumpkin peels hydrolysate, the dry weight of the pellet present at T_0_ was determined and subtracted to the value of the following measurements. The biomass and product yields were calculated as the ratio between the total amount of biomass or products and the amount of consumed sugars.

### Total lipid quantification

Lipid content was determined via the sulfo-phospho-vanilline colorimetric method (Spinreact, Spain) on the washed cell pellets corresponding to approximately 30 OD, suspended in 0.5 mL of cold redistilled water. The assays were performed in triplicate and standard deviations varied between 1 and 5%. Lipid yield was calculated as the ratio between total amount of product and amount of consumed carbon sources.

### Lipid profile analysis

The determination of fatty acid profile was carried out according to [51] and [52]. Briefly, a 2 g aliquot of the matrix was extracted according to [51] using first 7.5 mL of a 1:2 (v/v) solution of Chloroform:Methanol and shaking the sample. Next, 2.5 mL of 100% Chloroform and 2.5 mL milli-Q water were added, with further agitation of the sample after each solvent addition. Each sample was then centrifuged at room temperature for 10 min at 2000 rpm. The supernatant was then removed and, after filtration, evaporated by rotoevaporation in previously weighed flasks.

The extract obtained was resuspended in 1 mL of 95% hexane and an aliquot corresponding to 30 mg of fat was brought to dryness under a gentle nitrogen flow in a screw-capped tube for derivatization according to [52]. Briefly, the sample was incubated at 80 °C for 10 min after the addition of 0.5 mL of a Sodium Methoxide solution. Once cooled to 37 °C, 0.5 mL of a Bromine Trifluoride solution was added to the sample for the subsequent 3-min incubation at 80 °C to allow the formation of the methyl esters of the extracted fatty acids.

Methyl esters are extracted by adding 0.5 mL of 95% hexane and subjecting the sample to intense shaking for 1 min. After the addition of 1 mL of a saturated solution of Sodium Chloride and 100 mg of Sodium Sulfate Anhydrous, the sample is gently agitated and, after separation, the supernatant is removed and brought to dryness under a flow of Nitrogen. Finally, the sample is taken up with 1 mL of 98% Hexane and subjected to instrumental GC-FID analysis. The analysis of the methyl esters of the extracted fatty acids was carried out by gas chromatography using a gas chromatograph (TRACE GC Ultra, Thermo Fisher Scientific, Rodano, Italy) equipped with a flame ionisation detector (FID). An RT-2560 fused silica capillary column (100 m × 0.25 mm × 0.25 µm film thickness; Restek, Milan, Italy) was used with a temperature ramp programmed from 70 to 240 °C at 2 °C min^−1^. The carrier gas was nitrogen at 1.0 mL min^−1^ with an inlet pressure of 16.9 psi. A quantitative procedure was used in which 1 mL of internal standard (1 mg mL^−1^ 23:0 methyl ester; N-23-M; Nu-Chek Prep Inc., Elysian, MN, USA) was added prior to methylation. The content of fatty acid methyl esters (FAME) was quantified by weight expressed as g/100 g of total FAME. All analyses were performed in duplicate.

## Supplementary Information

**Additional file 1: Table S1.** Analysis of pumpkin peel hydrolysate obtained using 2.25 µL/mL of Cellic CTec2 for 24 h. **Table S2.** Complete *R. azoricus *fatty acid profile obtained in bioreactor after 90 h of process on pumpkin peel hydrolysate-based medium.

## Data Availability

The data sets used and/or analyzed during the current study are available from the corresponding author on reasonable request.
