# Supplementary material for: Recycling industrial food wastes for lipid production by oleaginous yeasts Rhodosporidiobolus azoricus and Cutaneotrichosporon oleaginosum
Source: Biotechnol Biofuels Bioprod. 2022 May 14;15:51. doi: 10.1186/s13068-022-02149-3 (PMC9107756; doi:10.1186/s13068-022-02149-3)
Supplement: Supplementary file 1 — Additional file 1: Table S1. Analysis of pumpkin peel hydrolysate obtained using 2.25 µL/mL of Cellic CTec2 for 24 h. Table S2. Complete R. azoricus fatty acid profile obtained in bioreactor after 90 h of process on pumpkin peel hydrolysate-based medium. [file 13068_2022_2149_MOESM1_ESM.docx]

**SUPPLENTANTARY MATERIALS**

**Table S1:** Analysis of pumpkin peel hydrolysate obtained using 2.25 µL/mL of Cellic CTec2 for 24 h.

|  | **Fraction1** | **Fraction2** | **Fraction3** | **Fraction4** |
| --- | --- | --- | --- | --- |
| **galactose (mg/g)** | 0 | 0 | 0 | 0 |
| **cellobiose (mg/g)** | 0 | 0 | 0 | 0 |
| **arabinose (mg/g)** | 0 | 0 | 5,33±1,81 | 0 |
| **DP4 (mg/g)** | 74,55±3,44 | 1,44±0,11 | 0 | 0 |
| **saccarosio (mg/g)** | 114,60±23,23 | 0 | 0 | 0 |
| **glucose (mg/g)** | 192,82±22,58 | 25,62±4,05 | 0 | 0 |
| **fructose (mg/g)** | 61,96±10,08 | 11,62±3,80 | 0 | 0 |
| **galatturonic acid (mg/g)** | 66,83±5,13 | 2,14±0,16 | 0 | 0 |
| **glucuronic acid (mg/g)** | 53,79±1,10 | 2,65±0,81 | 0 | 0 |
| **total** | 763,24±15,78 | 75,48±7,97 | 5,33±1,81 | 0 |
| **% on total** | 90,42±1,05 | 8,94±0,89 | 0,62±0,20 | 0 |

**Table S2:** Complete *R.azoricus* fatty acid profile obtained in bioreactor after 90 hours of process on pumpkin peel hydrolysate-based medium.

| **Fatty acid** | **Relative aboundance [%]** |
| --- | --- |
| C6:0 | 0,00 |
| C8:0 | 0,00 |
| C10:0 | 0,00 |
| C11:0 | 0,00 |
| C12:0 | 0,01 |
| C13:0 | 0,00 |
| C14:0 | 0,83 |
| C14:1 | 0,00 |
| C15:0 | 0,02 |
| C15:1 | 0,00 |
| C16:0 | 20,65 |
| C16:1 | 0,44 |
| C17:0 | 0,12 |
| C17:1 | 0,03 |
| C18:0 | 7,15 |
| C18:1n9t | 0,07 |
| C18:1n9c | 49,53 |
| C18:2n6t | 0,00 |
| C18:2n6c | 16,69 |
| C20:0 | 0,41 |
| C18:3n6 | 0,00 |
| C20:1 | 0,05 |
| C18:3n3 | 3,03 |
| C21:0 | 0,00 |
| C20:2 | 0,00 |
| C22:0 | 0,61 |
| C20:3n6 | 0,00 |
| C22:1n9 | 0,00 |
| C20:3n3 | 0,00 |
| C20:4n6 | 0,00 |
| C22:2 | 0,00 |
| C24:0 | 0,37 |
| C20:5n3 | 0,00 |
| C24:1 | 0,00 |
| C22:6n3 | 0,00 |
